# An analysis of contributing factors of head and neck space infections of odontogenic origin: A long-term retrospective clinical study (including COVID-19 pandemic period)

**DOI:** 10.4317/medoral.26018

**Published:** 2023-06-18

**Authors:** Junho Jung, Yeonjin Oh, Seung-jin Cha, Joo-Young Ohe

**Affiliations:** 1 Ph.D MSD DMD, Dept. of Oral and Surgery, School of Dentistry, Kyung Hee University, Seoul, Korea; 2 DMD, Dept. of Oral and Surgery, School of Dentistry, Kyung Hee University, Seoul, Korea

## Abstract

**Background:**

The purpose of this study is to investigate predisposing factors for the head and neck infections (HNIs), regarding to the demographic data, anatomical spaces, microbiology and antibiotic sensitivity for affected patients.

**Material and Methods:**

A 13-year of retrospective study evaluating 470 patients with HNIs, treated as inpatient management in the Department of Oral and Maxillofacial Surgery of KyungHee University school of Dentistry, Seoul, Korea, from January 2009 to February 2022. Statistical analysis of demographic, time-related, anatomic, microbiologic, and treatment variables were investigated for each patient.

**Results:**

The frequency of HNIs was significantly higher in 50’s in males, followed by 70’s in females. High Severity score (SS) were significantly associated with increased LOH (Length of hospital stay) and LOM (Length of medication), while LOH showed more intensive relationship compared with LOM. The most frequently involved space in abscess was submandibular space, though incidence and severity of HNIs shows declining tendency throughout 13-year research. *Streptococcus viridans* was the most predominant species isolated from pus culture growth, and a combination of ampicillin and sulbactam was the 1st choice of antibiotics intravenously. According to the comparison analysis between recommended antibiotics from resistance testing result and clinically administered antibiotics, final coincidence rate was estimated about 55%.

**Conclusions:**

Due to HNIs being multifactorial, predicting progression and management of HNIs is still a challenge for oral and maxillofacial surgeons. The present study showed several predisposing factors of SHNIs and their correlations, which could contribute to earlier diagnosis and more effective treatment planning for clinicians, thereby leading to the improvement of prognosis for patients, ultimately.

** Key words:**Head and neck infections (HNIs), severity score (SS), length of hospital stay (LOH), length of medication (LOM).

## Introduction

Infection is a disorder caused by an imbalance between humans and germs, and treatment of infection is to restore the balance between them by destroying the environment of germs and increasing the host's defense mechanism. As the oral cavity, which is the gateway to food intake in daily life, always has numerous germs, infection control in dental clinic has an important meaning.

Fortunately, there had been a significant decrease in incidence of head and neck space infections of odontogenic origin (HNIs) because of in the oral and maxillofacial region, immune function is developed by abundant blood circulation and since the surgical drainage technique developed and broad-spectrum antibiotics discovered. However, life-threatening complications such as mediastinitis, thoracic empyema, pericarditis, or septic shock with general multi-organ failure can occur, especially for untreated patients with associated systemic diseases (1). In addition, odontogenic infection in debilitated patients with systemic diseases such as heart disease may spread to a long distant metastasis and cause fatal infection or permanent disability.

About 70% of cases of HNIs are known as odontogenic in origin (2). Bacteria that cause odontogenic infections are mostly part of the resident flora of the host's oral cavity. These bacteria form colonies and are found in the gingival sulcus or oral mucosa, and are mainly aerobic gram-positive cocci, anaerobic gram-positive cocci, and anaerobic gram-negative bacilli. It causes odontogenic infection in adjacent tissues (2). Since the discovery of penicillin, the first antibiotic substance by Sir Alexander Fleming in 1928 which significantly changed the management of odontogenic infections, the research and development of numerous antibiotics which has different classes and therapeutic mechanisms have been founded (3). Although the occurrence of resistance to antibiotics has been frequently reported in many studies, no significant change has been revealed in the microbiology and antibiotic sensitivity of head and neck space infections over the last 30-40 years (4). Meanwhile, penicillin still remains the first choice of drug for their safe use, minimal side effects, and broad spectrum, especially in combination with beta-lactamase inhibitors (5).

In this approach to treatment of odontogenic infection, understanding the causative bacteria of odontogenic infection and knowing the mechanism of occurrence of odontogenic infection and the anatomical structure that can spread during infection are helpful in determining a treatment plan. In addition, it is necessary to know the principles of treatment for infected patients visiting the dentist in order to receive appropriate treatment. The purpose of the present retrospective study was to clarify the important indicators in treating HNIs including the backgrounds, the spaces involved by abscess, microbiology and antibiotic sensitivity for affected patients.

## Material and Methods

- Patients

A total of 470 patients of HNIs were included in this retrospective study, who were treated as inpatient management in the Department of Oral and Maxillofacial Surgery at the Kyung Hee University Hospital in Seoul, Korea, between January 2009 and August 2022. Hospitalized patients with diagnosis of HNIs of all age groups and both genders were included. Otherwise, those patients without taking 3-dimensional computed tomography angiography (CTA) on infected site and those with infection secondary to osteomyelitis or malignancy were excluded from the study. All the patients underwent surgical incision and pus drainage by intra-oral and/or extra-oral approach and routine IV treatment consisted of antibiotics and analgesics were administrated during hospitalization. This study was approved by the Clinical Research Ethics Committee of Kyunghee University Dental Hospital (Institutional Review Board KH-DT20031).

- Data collection

Clinical, radiographic, and laboratory parameters were evaluated through chart review, 3-dimensional computed tomography angiography (CTA) analysis, and the results of pus culture growth. The demographic variables were recorded comprising the patient’s age, gender and incidence of HNIs. The time-related variables such as length of hospital stay (LOH) and length of medication (LOM) were assessed for the purpose of evaluating the relationship between the treatment period and the severity of the abscess. To compare this, the Pearson correlation method was used.

The anatomic variables included the site of infected spaces according to the clinical diagnosis and analysis result of head and neck CTA. All fascial spaces involved in each patient were assigned to the severity score (SS) (6), based on the anatomic proximity to the odontogenic origins and extension range to the vital structures, such as airway or mediastinum. A severity score was calculated as the sum of all the spaces involved by abscess or cellulitis in a given patient. (SS: Buccal space(B), Submental space(SE) & Canine(C): 1, Temporal space(T), Infratemporal space(IT), Pterygomandibular space(PM), Sublingual space(SL), Submandibular space(SM) & Submasseteric space(ST) : 2, Parapharyngeal space(PP), Retropharyngeal space(RP) & Prevertebral space(PV) : 3)

The microbiologic variables and related antibiotics sensitivity were evaluated through the results of pus culture growth obtained by surgical drainage. From all 470 patients, a total of 177 cases of purulence were collected and sent to the pathology department of our hospital to identify the characteristics of microorganism, oxygen requirements (aerobic/anaerobic, obligate/facultative), and antibiotic susceptibility of the most leading pathogens through MIC (minimal inhibitory concentration) values. In several cases, even though multiple cultures taken during long period of hospitalization, the primary culture result was chosen for the statistical analysis to assess the valid strain and effective antibiotics at the onset of treatment.

Regarding to the treatment variables in this research, we focused on the 1st choice of antibiotics administered via intravenous route initially. Furthermore, the 2nd choice of antibiotics was also examined which was chosen for the purpose of improved treatment results if the former considered as invalid. Then, the comparison analysis was performed to estimate match rate between the antibiotics actually administered to the patient and the most susceptible antibiotics for the leading pathogen which was revealed from the pus culture growth and antibiotics sensitivity test (MIC) result.

The data were analyzed through Fisher’s exact, chi-square, t test, linear regression, Pearson’s correlation analysis and statistical analysis was performed using SPSS (SPSS version 25.0, Chicago, IL). P-value less than 0.05 was considered statistically significant.

## Results

- Demographic variables

**Figure 1:**
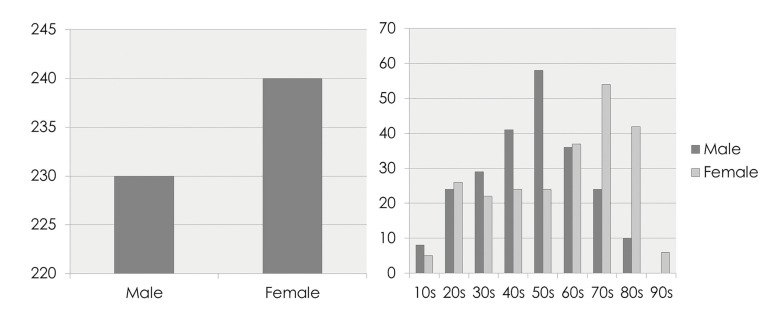
Distribution of age and gender.

Among 470 patients of HNIs, 230 (48.9%) male patients and 240 (51.1%) female patients were ranged in age from 6 to 99 years, with a mean age of 55.5 years old. The average age of males was 50.8 years, while that of females was 59.9 years. The most predominant age group was 50’s (12.3% of total) in males, followed by females of 70’s (11.5% of total) (Fig. 1). The correlation between diabetes mellitus (HbA1c>5.7) and treatment duration was analyzed. It was found that the higher the SS, the statistically significantly longer the LOH in diabetics (*p*<0.05) (Table 1).

- Time-related variables

Average length of hospital stay (LOH) was calculated as 7.4 days, whereas the average period of medication until the treatment ends (LOM) was 15.6 days. To investigate the relationship between the treatment period and the severity of the abscess, we obtained Pearson correlation coefficient (ρ) from severity score (SS) for affected spaces and length of hospital stay (LOH) or length of medication (LOM) for each patient (Table 2). The data suggested that there were positive correlation either between SS and LOH (ρ=+0.611, *p*<0.05) or SS and LOM (ρ=+0.294, *p*<0.05), while LOH showed more strong correlation with SS than that of LOM with SS, relatively (Table 2).

**Table 1:**
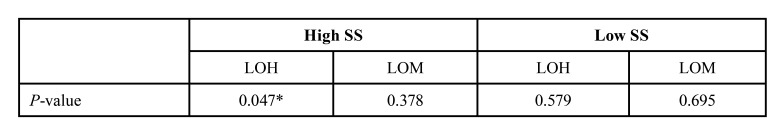
Correlation coefficient between severity score (SS) and diabetes mellitus (DM).

**Table 2:**
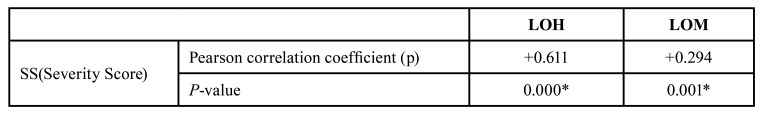
Correlation coefficient between severity score (SS) and length of hospital stay (LOH), length of medication (LOM).

- Anatomic variables

**Figure 2:**
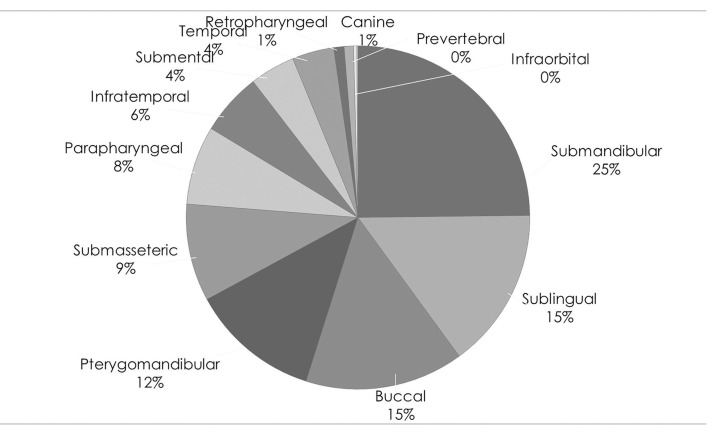
Distribution of affected spaces of HNIs.

According to 14 years of present study from 2009 to 2022, average number of affected spaces was shown to be 2.1±1.3, ranged from 1 to 8 spaces (Table 3). Average severity score was 3.6±2.8 in the range of 1-18. To compare the differences in the degree of infection caused by the improvement of the people's health concept after the COVID-19 pandemic, the analysis was divided into before and after the COVID-19 pandemic. As presented in Table 3, former 11 years of (2009-2019) statistical result shows that mean number of spaces affected was 2.3±1.5 and SS was 3.9±3.5. Meanwhile, the result for latter 3 years showed slight decrease values on both number (1.4±0.8) and SS (2.5±1.7). The most frequently involved space in abscess was submandibular space (25%) followed by sublingual (15%), buccal (15%) and pterygomandibular space (12%) (Fig. 2).

**Table 3:**
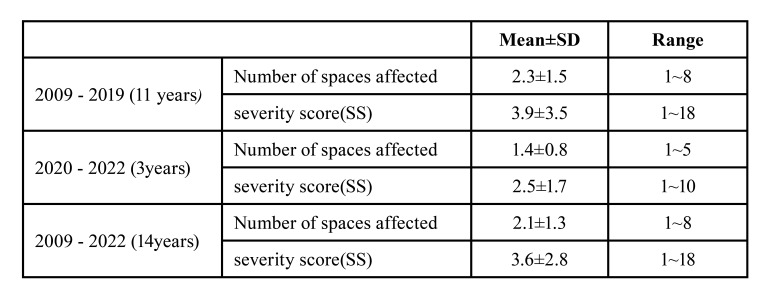
Average and range of number of affected spaces and severity score for 14 years retrospective study: from 2009 to 2019 years (former 11 years); from 2020 to 2022 years (later 3 years); from 2009 to 2022 years (total 14years).

**Figure 3:**
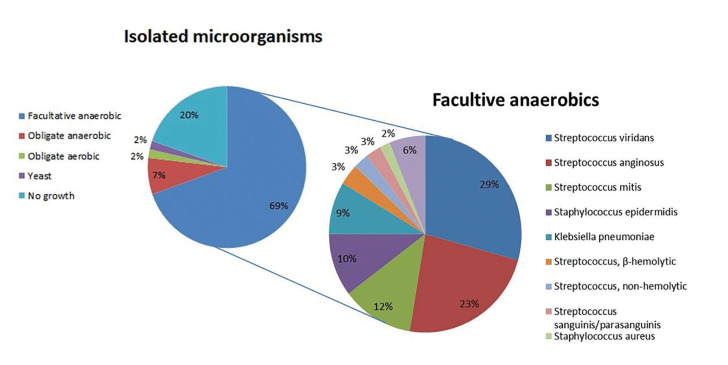
Distrubution of microorganisms.

- Microbiologic variables

For 470 HNIs patients, 177 pus cultures were taken for microbiological studies (Fig. 3). Though 35 cases revealed no growth of microorganism, 142 cases of microbiological isolates including 123 facultative anaerobes (69.5%), 13 obligate anaerobes (7.3%), 3 obligate anaerobes (1.7%) and 3 yeasts (1.7%) were identified. Out of the 123 isolates (facultive anaerobes), *Streptococcus viridans* was the most frequently found microorganism (29.2%), followed by *Streptococcus anginosus *(23.3%) and *Streptococcus mitis* (11.9%). *Strepcococci* is the major causative organism in infection of head and neck area (42.4%). *Staphylococcus epidermis* (10%) and *Klebsiella pneumoniae* (10 cases, 9%) followed. In obligate anaerobes, *Prevotella intermedia* (5 cases, 1.8%) was predominantly detected. Obligate aerobes also found in small percentage of isolates, including *Kluyvera ascorbata*, *Neisseria* spp, and *Pseudomonas fluorescens* (1 case, 0.3%, each).

- Treatment variables

**Figure 4:**
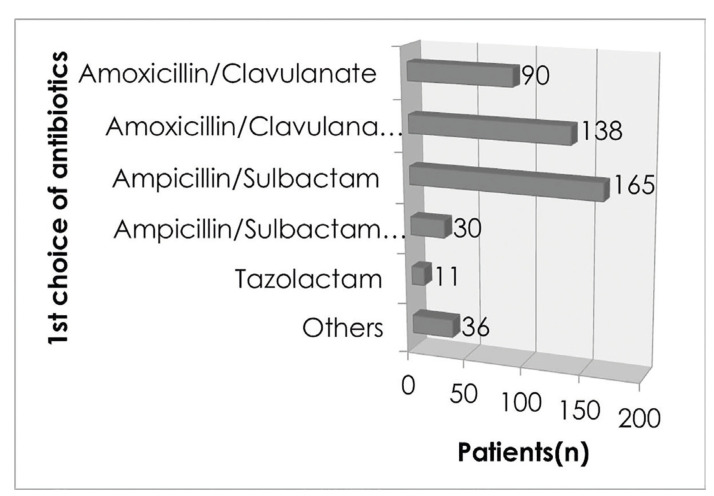
Distribution of 1st choice of antibiotics.

A total of 470 patients were given antibiotics via IV and appropriate surgical drainage was performed. The most common choice of initial antibiotics was a combination of ampicillin and sulbactam (165/470 cases), followed by amoxicillin/clavulanate concomitant with isepamacin or metronidazole or tazolactam (138/470 cases) (Fig. 4). Some patients who received single dose of tazolactam (11/470 cases), or other drugs such as clindamycin, levofloxacin or ciprofloxacin (36/470 cases) were found to be in minor groups relatively. Antibiotic sensitivity testing was performed only for 107 cases of leading pathogens among 177 valid isolates of microorganism. Table 4 shows the rate of coincidence between recommended antibiotics and clinically administered antibiotics: Recommended antibiotics (number of cases) from sensitivity test which shows the Lowest MIC value; Initial choice of IV antibiotics (number of cases) clinically administered to relevant patient before obtaining microbiological assay result; final choice of IV antibiotics (number of cases) clinically administered to relevant patient after obtaining microbiological assay result.

**Table 4:**
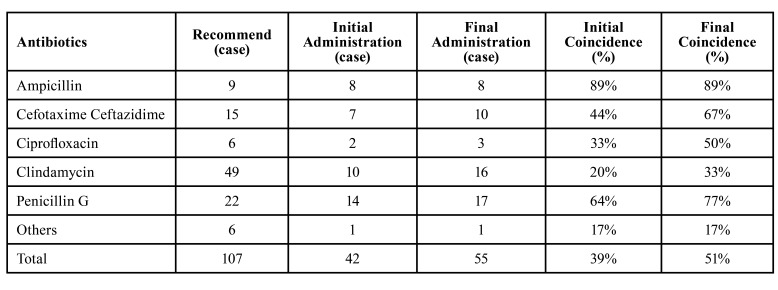
The rate of coincidence between recommended antibiotics and clinically administered antibiotics.

Initial coincidence rate (%) between antibiotics recommended and administered; Final coincidence rate (%) between antibiotics recommended and administered actually. Regarding to the sensitivity/resistance assay results, the susceptible antibiotics that show the lowest MIC values were counted by type of drugs (Table 4). Meanwhile, the initial choice of IV antibiotics clinically administered to each patient before obtaining the results for sensitivity testing was counted. After gaining the testing results for few days later, the final choice of IV antibiotics administered to relevant patient was shown at Table 4. Then, the results of this analysis were used to compare to identify the rate of coincidence between recommended and clinically administered antibiotics. As the result of comparison of recommend antibiotics with initial antibiotics, total initial coincidence rate was 39%. Comparing recommend antibiotics with final antibiotics, final coincidence rate was demonstrated as 51% entirely, showing slight improvement of concordance level.

## Discussion

Head and neck space infections (HNIs) are still frequently occurred presentations within the maxillofacial area, requiring an immediate and accurate treatment at most (7). In the majority of cases, it is sufficient to deal with HNIs with the three keys as below: 1) protection and control of the airway, 2) antibiotic therapy, and 3) surgical drainage of the abscess (8). Nevertheless, the need for safe, effective and successful treatment is still exist, which would be established by fully understanding our patients’ characteristics, pathology, microbiology, and directions of recent treatments entirely. So, this study analyzed the correlation of various factors with the distribution of the HNIs.

In the analysis for demographic variables, there was no predominance on gender, showing 48.9% for male and 51.1% for female patients. These findings are consistent with previous studies (7,9). Although these studies identified slight male predilection (54% for males, 46% for females), no statistical difference was revealed. The mean age of HNIs was 55.5 years old, younger in males (50.8 years) and much older in females (59.9 years) respectively. And the 50’s in male had the highest frequency of HNIs (12.3%), followed by females of 70’s (11.5%). This finding was similar to the results of other previous studies (9,10). There are some previous studies indicating the etiology of HNIs (11,12). Diabetes mellitus, smoke, alcohol or drugs users are more likely to show poor oral hygiene and the factors are pointed out as a predisposition of deep neck infection. In this study, the correlation between diabetes mellitus (HbA1c>5.7) and treatment duration was analyzed. It was found that the higher the SS, the statistically significantly longer the LOH in diabetics (*p*<0.05). This was similar to previous studies (12,13).

The average LOH was 7.4 days, and LOM was 15.6 days, including the longer period after discharge from hospital until the medication and treatment ends. It means that after discharge, about a week of additional medication is required to treat HNIs. SS and LOH (ρ=+0.611, *p*<0.05) showed more strong correlation than SS and LOM (ρ=+0.294, *p*<0.05), indicating the severity of the abscess is positively related to the period of hospitalization rather than total medication period. The reason is estimated that more severe head and neck infection needs more intensive care, airway management and IV administration while staying in hospital.

Regarding the anatomic variables, average number of affected spaces was 2.1±1.3 (range of 1-8 spaces), which means it frequently happened as multiple space infection, two or more spaces involved concurrently. The most frequently affected space was submandibular space (25%), which correlated to the study by Walia *et al*. in 2014 (14). To compare the differences in the degree of infection caused by the improvement of the people's health concept after the Covid-19 pandemic, the analysis was divided into before and after the Covid-19 pandemic. From 2009 to 2019 (former 11 years), mean number of spaces was 2.3±1.5 and SS was 3.9±3.5. By comparison, from 2019 to 2022 (the latter 3 years), the average number of spaces was 1.4±0.8 and the SS decreased to 2.5±1.7. This is attributed to hand washing, social distancing, increased awareness of oral health and improved oral hygiene due to Covid-19 in the last 3 years (15).

Pus cultures were taken for 177 out of 470 patients, and 142 isolates being identified from microbiology assay. The majority of microorganisms were classified as facultative anaerobes (69.5%) and *Streptococcus viridans* (19.2%) were the most predominant pathogen. This finding is directly corresponding to previous literature reviews (7,16-18). *Staphylococci* (7.9%) took over the second genus including *Staphylococcus epidermis* (6.8%) and *Staphylococcus aureus* (1.1%), followed by *Klebsiella pneumoniae* (5.6%). The reason why the species are predominant in HNIs may be due to the fact that the high rate of odontogenic origin is Figured out as main cause of HNIs (19,20).

Most of cases in Department of Oral and Maxillofacial Surgery at the Kyung Hee University Hospital, 2 broad-spectrum antibiotics were used usually. The first choice of drug was ampicillin with sulbactam, and the second was amoxicillin with clavulanate, both were combinations of a semisynthetic derivative of penicillin and β-lactamase inhibitor. Actually, it is known that clavulanate could effectively neutralize most of beta-lactamases released from the odontogenic infection-related bacteria (21). On the other hand, the overall resistance rate of bacteria are still rising and the majority of HNIs are polymicrobial, clinicians should consider alternative antibiotic options adjusted by pus culture and sensitivity testing results (8). In our study, we tried to Figure out the adjustment of antibiotics before/after obtaining the sensitivity testing result by comparing the initial and final coincidence rate of recommended and clinically administered antibiotics. As seen in our review, antibiotics initially administered before culture result gaining was mostly broad-spectrum antibiotics of 1st choice, which showed 39% of coincidence rate with the recommended antibiotics obtained from sensitivity testing found later. After acquiring the culture result, the final coincidence rate was 51%, which means 2nd or 3rd choice of antibiotics were adjusted to fit pus culture results in only 12% of cases. This result may due to the fact that the antibiotics initially used were effective enough in improving the symptoms so that clinicians did not change the drug intentionally, or that the inflammatory symptoms improved rapidly and the patient was discharged before confirming the pus culture results. To clarify this more clearly, further research may be necessary, which is a comparative follow-up study between a group that immediate change antibiotics regarding to the pus culture result obtained and the other group that maintain the existing effective antibiotics instead of changing it, prospectively.

The main limitations of present study comprise the absence of important information regarding patients’ medical conditions and various comorbidities (e.g., diabetes mellitus, smoking, immune-deficient diseases) with known impact on infection emergence (22), progression and prognosis (23). As HNIs remain a challenging and emergency disease for oral and maxillofacial surgeons, stronger acknowledgement of etiology, curious attention to disease progression and continuous effort to reach an appropriate clinical and therapeutic management should be taken into consideration, thorough.
